# Bilateral Choroidal Metastases Mimicking Central Serous Chorioretinopathy: A Diagnostic Challenge

**DOI:** 10.7759/cureus.91842

**Published:** 2025-09-08

**Authors:** Ricardo A Murati Calderon, Christian Nieves-Ríos, Luis F Nieves Garrastegui, Luis D Flores Cabán, Armando Oliver

**Affiliations:** 1 Ophthalmology, School of Medicine, Medical Sciences Campus, University of Puerto Rico, San Juan, PRI; 2 Pulmonology, Hospital San Francisco, San Juan, PRI; 3 Oncology and Hematology, Hospital San Francisco, San Juan, PRI

**Keywords:** central serous chorioretinopathy, choroidal metastasis, multimodal ophthalmic imaging, ocular oncology, pulmonary squamous cell carcinoma

## Abstract

We report a case of bilateral choroidal metastases initially misdiagnosed as central serous chorioretinopathy (CSC) in a Hispanic man with no prior oncologic diagnosis. A 72-year-old Hispanic man with no history of ocular disease was referred to our clinic for evaluation of bilateral serous retinal detachments presumed to be CSC. His symptoms, including progressive blurry vision in both eyes, had developed over several months without spontaneous resolution. Multimodal imaging, including fundus autofluorescence (FAF), fluorescein angiography (FA), optical coherence tomography (OCT), and B-scan ultrasonography, revealed bilateral subretinal lesions and atypical features inconsistent with CSC. Given these findings and the patient’s report of recent hemoptysis, a systemic workup was pursued, revealing a spiculated mass in the left upper lobe. Biopsy confirmed pulmonary squamous cell carcinoma, staged as T2aN1M1 (Stage 4B). The patient was treated with chemotherapy, thoracic external beam radiotherapy, and bronchodilator therapy; however, upon follow-up, the choroidal lesions and serous retinal detachments worsened, and visual acuity did not improve. This case demonstrates the importance of including choroidal metastases in the differential diagnosis of bilateral serous retinal detachments, especially in older patients or those with systemic red flags. It highlights the crucial role of ophthalmologists in identifying undiagnosed systemic malignancies through fundus examination and multimodal imaging.

## Introduction

Choroidal metastasis represents the most common intraocular malignancy in adults, often originating from a primary breast or lung carcinoma [1]. Within the eye, the choroid is the primary site for metastatic tumor deposition due to its rich vascular supply [2]. Choroidal metastatic lesions typically present as yellow-white subretinal masses associated with overlying serous retinal detachment and are most often located in the posterior pole [2,3]. Although unilateral involvement is more common, bilateral cases have been reported in 20%-40% of patients, particularly those with widespread systemic disease [2-4]. While ocular involvement generally occurs late in the course of metastatic disease, in rare instances, choroidal metastasis may present as the initial manifestation of systemic malignancy, posing a significant diagnostic challenge [3].

Despite their relatively high frequency among intraocular tumors, choroidal metastases may present with subtle clinical features that can mimic benign retinal conditions, most notably central serous chorioretinopathy (CSC) [5]. Characterized by idiopathic serous detachment of the neurosensory retina, CSC typically affects young and middle-aged men, often in association with psychological stress or corticosteroid use [6,7]. Both CSC and choroidal metastasis can present with painless visual disturbances and subretinal fluid, sometimes without overt systemic symptoms [8].

This clinical overlap can lead to diagnostic delays. Several case reports have described choroidal metastasis that was initially misdiagnosed as CSC, particularly when the lesions were amelanotic, poorly demarcated, and/or associated with localized subretinal fluid [5,9]. Multimodal imaging, including fundus autofluorescence (FAF), fluorescein angiography (FA), optical coherence tomography (OCT), and B-scan ultrasonography, is crucial in distinguishing between these entities [8]. Enhanced-depth imaging OCT may reveal key features of choroidal metastasis, such as choroidal undulations, posterior shadowing, and irregular hyperreflectivity [10].

In this report, we describe a diagnostically challenging case of bilateral choroidal metastases in a 72-year-old man who was initially misdiagnosed with CSC. Further investigation with a systemic workup revealed a previously undiagnosed primary pulmonary squamous cell carcinoma. This case emphasizes the importance of a broad differential diagnosis when evaluating serous retinal detachments, highlights the utility of multimodal imaging in distinguishing benign from malignant retinal pathologies, and reinforces the pivotal role of ophthalmologists in identifying systemic malignancies through ocular findings.

## Case presentation

A 72-year-old Hispanic man was referred by a local ophthalmologist to our clinic for evaluation of bilateral serous retinal detachments observed on initial fundus examination, with a preliminary impression of CSC. The patient reported progressive blurry vision in both eyes (OU) over several months. He denied any prior ocular diagnoses or procedures; however, his medical history was notable for a previously identified pulmonary mass, for which he had discontinued diagnostic evaluation for personal reasons. He also reported a history of active tuberculosis diagnosed over 45 years earlier, which had been appropriately treated and had not recurred. The review of systems was negative for fever, chills, arthralgia, skin rash, imbalance, or headache, and he denied any recent travel. He did report unintentional weight loss and a recent episode of hemoptysis two weeks before presentation. There was no significant family history. His social history was remarkable for smoking for over 58 years.

On comprehensive ophthalmic evaluation, his best-corrected visual acuity was 20/60 in the right eye (OD) and 20/200 in the left eye (OS). Intraocular pressure by applanation was 16 mmHg OU. Pupils were round and reactive to light, with no afferent pupillary defect. Extraocular movements were full in all directions of gaze OU, without pain. Confrontation visual fields were full to finger counting in all quadrants OU. Color vision testing with Ishihara plates was normal in OD (14/14) but revealed dyschromatopsia in OS (3/14). External examination and slit-lamp examination revealed no significant findings other than 2+ nuclear sclerosis in OU; there were no cells or flare in the anterior chamber or vitreous.

**Figure 1:**
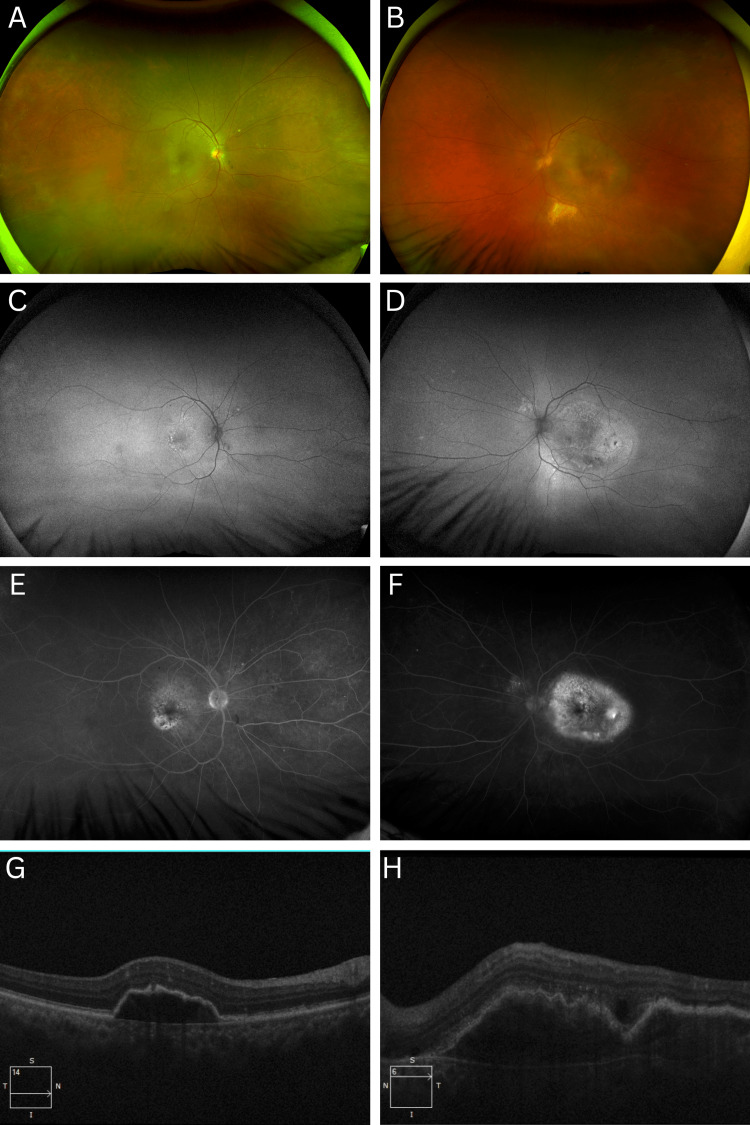
Ultra-widefield fundus imaging and optical coherence tomography of the right and left eye at presentation (A) Color fundus photograph of the OD shows subtle hypopigmented subfoveal choroidal lesions. (B) Left eye demonstrates a serous macular detachment with more pronounced submacular hypopigmented lesions and hard exudates along the inferior arcades. (C) Fundus autofluorescence of OD reveals multiple hyperautofluorescent spots temporal to the fovea and central macular hypoautofluorescence. (D) Fundus autofluorescence of OS shows macular hyperautofluorescence in a gravity-dependent pattern with a central geographic area of hypoautofluorescence. (E) Fluorescein angiography of OD shows macular transit defects and focal staining inferotemporal to the macula. (F) Fluorescein angiography of OS reveals a well-circumscribed area of staining within the macula. (G) High-resolution OCT of OD shows a dome-shaped elevation of the retinal pigment epithelium (RPE) with undulated borders and predominantly hyporeflective, mixed reflectivity. (H) OCT of OS reveals a dome-shaped choroidal lesion with undulated borders, mixed echogenicity, and an associated inferior retinal detachment.

Initial dilated fundus examination of OD (Figure 1A) revealed subtle hypopigmented subfoveal choroidal lesions. OS (Figure 1B) demonstrated a serous macular detachment with more prominent submacular hypopigmented lesions and hard exudates distributed along the inferior arcades. Fundus autofluorescence imaging of OD (Figure 1C) showed multiple hyperautofluorescent spots temporal to the fovea and a diffuse area of central hypoautofluorescence. In contrast, OS (Figure 1D) exhibited macular hyperautofluorescence in a gravity-dependent pattern, accompanied by a geographic area of central hypoautofluorescence.

Fluorescein angiography of OD (Figure 1E) revealed macular transit defects and a focal area of staining inferotemporal to the macula. OS (Figure 1F) showed a well-circumscribed area of staining within the macula. High-resolution OCT of OD (Figure 1G) demonstrated a dome-shaped choroidal lesion elevating the retinal pigment epithelium (RPE) with undulated borders and predominantly hyporeflective, mixed internal reflectivity. Similarly, OCT of OS (Figure 1H) revealed a dome-shaped choroidal lesion elevating the RPE with similar undulated borders and mixed echogenicity, along with an associated retinal detachment extending along the inferior margins. B-scan ultrasonography showed hyperreflective choroidal lesions with acoustic shadowing in OU.

**Figure 2:**
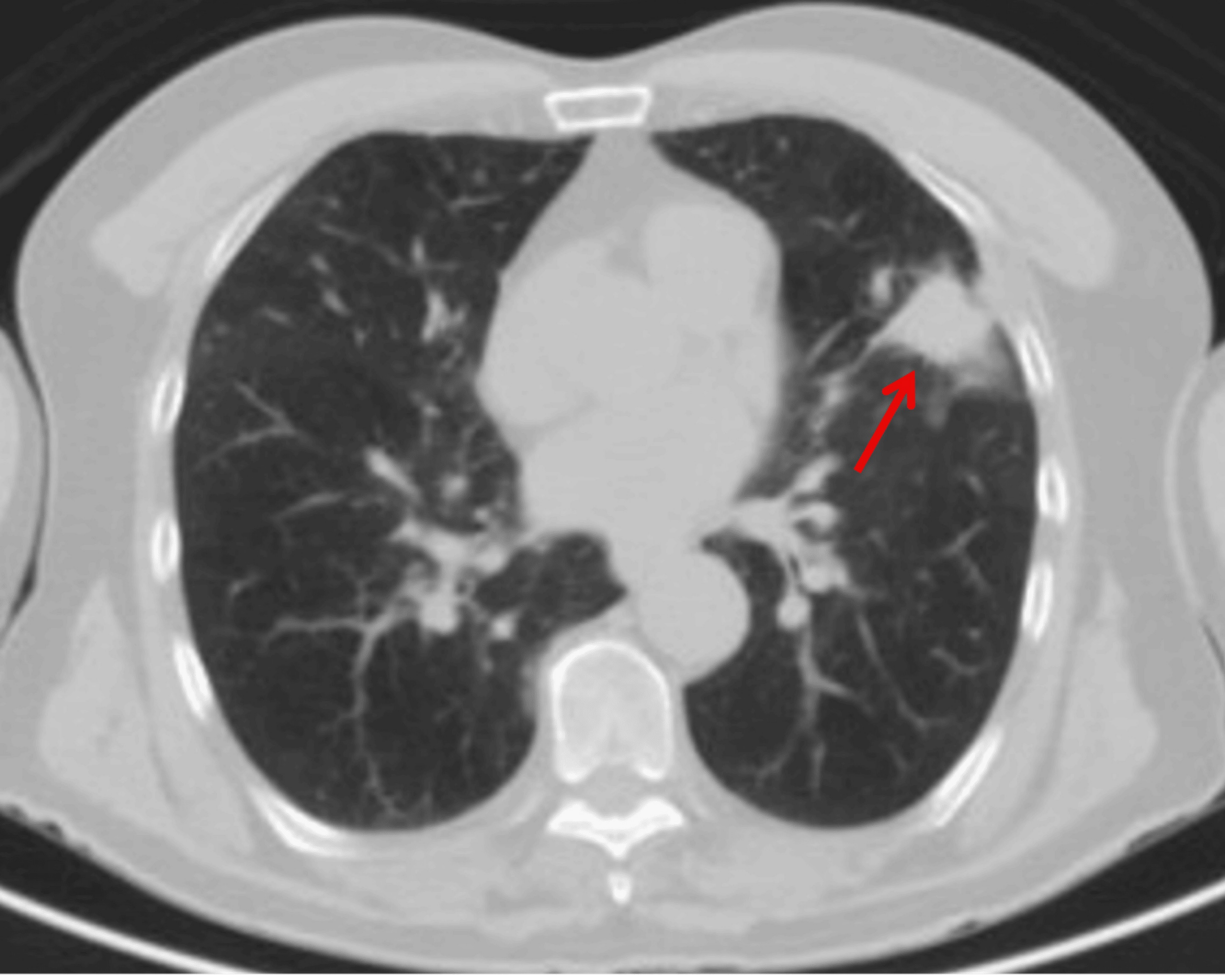
Chest CT showing a spiculated pulmonary mass in the left upper lobe Axial chest computed tomography (CT) scan demonstrates a spiculated mass (red arrow) in the lingular segment of the left upper lobe, measuring 3.0 × 2.4 × 1.7 cm. Surrounding moderate emphysematous changes are also noted.

**Figure 3:**
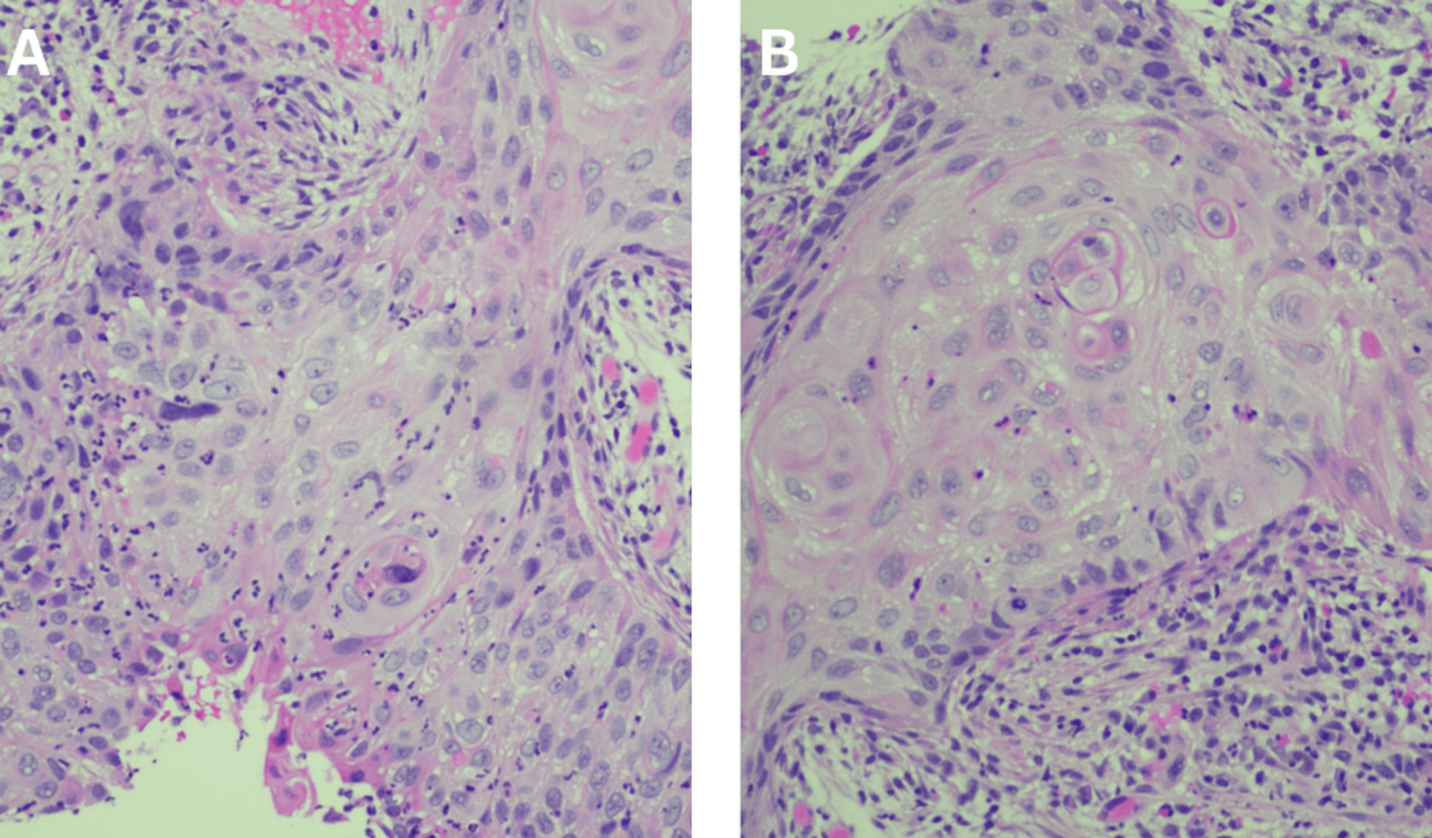
Histopathology of the primary pulmonary lesion (H&E) (A and B) Well-differentiated, keratinizing squamous cell carcinoma with invasive nests showing intercellular bridges and prominent keratin pearls (H&E, 20x). H&E: hematoxylin and eosin.

A presumptive diagnosis of bilateral choroidal metastasis was made based on the ocular findings and the patient’s prior history of a suspected pulmonary mass. He was referred to a local pulmonologist and an oncologist for a comprehensive systemic evaluation. Chest computed tomography (CT) (Figure 2) revealed moderate emphysematous changes and a spiculated mass in the lingular segment of the left upper lobe measuring 3.0 × 2.4 × 1.7 cm. Laboratory evaluation, including a complete blood count and a metabolic panel, was largely unremarkable aside from elevated serum bicarbonate levels (CO₂: 30.7 mmol/L), suggestive of chronic hypercapnia. A fine-needle aspiration biopsy of the pulmonary lesion confirmed the diagnosis of squamous cell carcinoma, staged as T2aN1M1 (stage 4B) (Figure 3).

The patient was initiated on bronchodilator therapy with albuterol every eight hours, along with systemic chemotherapy, and subsequently underwent 10 sessions of external beam radiotherapy to the primary lesion. He reported subjective improvement in his respiratory symptoms during follow-up visits. A whole-body positron emission tomography-CT scan was scheduled to assess for further metastatic spread.

After being lost to follow-up, the patient returned for evaluation 10 months after the initial presentation. At that time, his visual acuity had declined to counting fingers in both eyes. Fundus examination revealed progression of the choroidal lesions bilaterally, with the development of a serous retinal detachment in the right eye. Given the worsening clinical course and characteristic findings of metastatic disease, the patient was referred to an ocular oncologist for further management.

## Discussion

In this report, we present a unique case that highlights a rare presentation of bilateral choroidal metastases as the first clinical manifestation of an undiagnosed pulmonary squamous cell carcinoma. While lung cancer is a known source of ocular metastasis, most reported cases are unilateral and occur after a systemic malignancy has already been diagnosed. In a retrospective cohort of 70 patients with choroidal metastasis, Blasi et al. reported bilateral involvement in only 29% and found that just 14% appeared to be cancer-free at the time of ocular presentation; all were later confirmed to have lung cancer [4]. Similarly, Tsutsumi et al. reported that 43% of their patients with intraocular or ocular adnexal metastases had no prior cancer history, reinforcing the diagnostic importance of ocular findings in the detection of systemic malignancy [3].

Our patient was initially misdiagnosed with CSC, a condition typically affecting younger men and associated with stress or steroid use [11]. Although CSC and choroidal metastasis differ in etiology and prognosis, both can present with serous retinal detachment and visual disturbances. In their review, Sahoo et al. emphasized CSC as one of the most commonly misdiagnosed retinal disorders, particularly in bilateral or atypical cases with subretinal exudation, irregular pigment epithelial detachments (PEDs), or absence of spontaneous resolution [9]. Van Dijk et al. further noted that a variety of posterior segment pathologies, including certain neoplastic and inflammatory conditions, can mimic CSC, highlighting the critical role of multimodal imaging in establishing an accurate diagnosis [5].

In this case, multimodal imaging was instrumental in revising the initial diagnosis from CSC to solid choroidal tumors. Specifically, on OCT, both eyes showed dome-shaped choroidal masses elevating the RPE with heterogeneous internal reflectivity and shallow subretinal fluid, findings that indicate an infiltrative lesion rather than PEDs characteristic of CSC. On FAF, there was localized, sharply demarcated central hypoautofluorescence overlying the lesions, a pattern consistent with blockage from an elevated choroidal mass; notably, the granular or mottled changes typical of chronic CSC were absent. Fluorescein angiography revealed macular transit defects and focal staining in OD, as well as a well-circumscribed area of macular staining in OS, further supporting the diagnosis of choroidal metastases. Additionally, B-scan ultrasonography demonstrated acoustically dense, solid lesions, findings not expected in CSC, which lacks a discrete mass [12].

The systemic workup in our case revealed a spiculated mass in the left upper lobe, ultimately diagnosed as squamous cell carcinoma, a less common subtype associated with ocular metastases than adenocarcinoma. In a systematic review by Singh et al., adenocarcinoma was reported as the most common pulmonary malignancy leading to choroidal metastases. In contrast, squamous cell carcinoma accounted for a minority of cases [13]. This aligns with a report by Salah et al., which described a case of bilateral choroidal metastases secondary to lung adenocarcinoma in which ocular symptoms were the first sign of systemic malignancy [14]. Although rare, such presentations underscore the need for high clinical suspicion of metastatic disease, particularly in older patients or those with systemic red flags such as weight loss or hemoptysis.

The treatment of choroidal metastases typically targets the primary malignancy [8]. In our case, the patient received a chemotherapy regimen consisting of gemcitabine plus cisplatin, thoracic EBRT (external beam radiation therapy), and bronchodilator therapy. Despite treatment, the serous retinal detachments and choroidal masses progressed, and his visual acuity worsened. This outcome was consistent with prior reports noting that while EBRT or systemic therapy may control tumor progression, visual recovery is often limited due to irreversible retinal damage or subfoveal involvement [8,15]. These findings emphasize the importance of early detection and prompt intervention to optimize visual outcomes.

This case reinforces the importance of considering metastatic disease in the differential for serous retinal detachments, particularly in elderly patients with systemic symptoms or an incomplete cancer workup. It underscores how ophthalmologists, through early detection and systemic referral, play a critical role in uncovering underlying malignancies.

## Conclusions

This case underscores the diagnostic challenges posed by bilateral serous retinal detachments in the absence of a known malignancy. The initial misdiagnosis of CSC highlights how atypical imaging findings can obscure the presence of underlying metastatic disease. It reinforces the importance of maintaining a broad differential diagnosis when evaluating serous retinal detachments, particularly in elderly patients or those with systemic red flags. Multimodal imaging and careful clinical correlation are critical for distinguishing malignant lesions from benign entities such as CSC. Prompt systemic evaluation in atypical presentations is essential for early diagnosis, timely oncologic referral, and improved patient outcomes.
